# Ex vivo rectal explant model reveals potential opposing roles of Natural Killer cells and Marginal Zone-like B cells in HIV-1 infection

**DOI:** 10.1038/s41598-020-76976-5

**Published:** 2020-11-19

**Authors:** S. Abigail Smith, Phillip M. Murray, Praveen Kumar Amancha, Cassie G. Ackerley, Yi-Juan Hu, Rama R. Amara, Colleen F. Kelley

**Affiliations:** 1 Division of Infectious Disease, The Hope Clinic of the Emory Vaccine Center, Emory University School of Medicine, Decatur, GA 30030 USA; 2 Departent of Biostatistics and Bioinformatics, Emory University, Atlanta, GA 30322 USA; 3 Emory Vaccine Center, Yerkes National Primate Research Center, Emory University, Atlanta, GA 30329 USA

**Keywords:** Virology, Immunology, Innate immune cells, Innate immunity, Mucosal immunology

## Abstract

Our understanding of innate immune responses in human rectal mucosal tissues (RM) and their contributions to promoting or restricting HIV transmission is limited. We defined the RM composition of innate and innate-like cell subsets, including plasmacytoid dendritic cells; CD1c + myeloid DCs; neutrophils; macrophages; natural killer cells (NK); Marginal Zone-like B cells (MZB); γδ T cells; and mucosal-associated invariant T cells in RM from 69 HIV-negative men by flow cytometry. Associations between these cell subsets and HIV-1 replication in ex vivo RM explant challenge experiments revealed an inverse correlation between RM-NK and p24 production, in contrast to a positive association between RM-MZB and HIV replication. Comparison of RM and blood-derived MZB and NK illustrated qualitative and quantitative differences between tissue compartments. Additionally, 22 soluble molecules were measured in a subset of explant cultures (n = 26). Higher production of IL-17A, IFN-γ, IL-10, IP-10, GM-CSF, sFasL, Granzyme A, Granzyme B, Granulysin, and Perforin following infection positively correlated with HIV replication. These data show novel associations between MZB and NK cells and p24 production in RM and underscore the importance of inflammatory cytokines in mucosal HIV infection, demonstrating the likely critical role these innate immune responses play in early mucosal HIV replication in humans.

## Introduction

The rectal mucosa (RM) is a critical mucosal site in the transmission and pathogenesis of HIV-1 infection. Receptive anal intercourse carries the highest risk of HIV-1 acquisition among sexual exposures (1.38% per exposure event)^1^. It is thought that RM tissue imposes reduced physiological and immunological barriers to HIV transmission, relative to cervical and penile epithelium, due to the vulnerability of the single-layer columnar epithelium to micro-abrasions, as well as the relative abundance of CD4 + T cells within the RM^2,3^. Nonetheless, it is possible the selective pressures exerted by the RM are more restrictive and complex than previously assumed^4^. In the absence of an efficacious HIV-1 vaccine or widely accessible and utilized pre-exposure prophylactic interventions, defining the RM immune environment at the time of HIV-1 exposure and identifying the immune cell subsets restricting or contributing to HIV replication within the RM could inform strategies that seek to strengthen the barriers to HIV-1 transmission within this tissue.

Activated CD4 + T cells within the rectum have been associated with virus acquisition^5^, and the importance of CD4 + T cells is clear in mucosal transmission^2,3^. However, the roles of the diverse innate immune cells residing within RM tissue, which could pose potential obstacles to successful HIV transmission at this site, are less clear. Innate and innate-like immune cell subsets are capable of recognizing and responding to invading pathogens either directly, or through the identification and elimination of locally infected cells. Examples of direct inhibitory immune interactions include neutrophils, which can secrete Neutrophil Extracellular Traps that inactivate infectious virus, as well as antigen presenting cells (APC), such as macrophages and dendritic cells, which capture and process HIV for presentation and activation of T cells^6,7^. HIV infection can also be impeded through the recognition and elimination of infected cells by Natural Killer cells (NK), Mucosal associated invariant T cells (MAIT), and γδ T cells, which each express various anti-viral pattern-recognition receptors, such as Natural Cytotoxicity Receptors (NCR), Killer Cell Lectin-Like Receptors (KLR), and Killer Cell Immunoglobulin-Like Receptors (KIR)^8–10^. Furthermore, these innate cells also secrete a cadre of cytokines, such as Type I and Type II interferons, which could negatively influence HIV-1 replication^10,11^.

Conversely, RM-residing innate cell subsets could also contribute to productive HIV-1 infection. Both macrophages and dendritic cells are suitable targets for HIV infection^12^, and while APC play an essential role in shaping the adaptive immune response, they can also actively contribute to HIV-1 replication via *trans* infection^13^. That is, virions captured by APC remain intact on the cell surface, and these infectious virions are inadvertently presented directly to susceptible CD4 + T cells via the immunological synapse. In ex vivo models, macrophages, dendritic cells, and B cells isolated from peripheral blood are able to mediate *trans* infection, resulting in p24 accumulation orders of magnitude greater than levels associated with direct *cis* infection of CD4 + T cells^14,15^. This process could be highly relevant during mucosal transmission, where APC have been implicated as actors in the first stages of intravaginal infection^16,17^. Interestingly, a unique subset of B cells that are considered ‘innate-like’, Marginal Zone-like B cells (MZB), are also found within mucosal and epithelial barriers in humans, including the RM, and have been recently associated with HIV transmission and pathogenesis in humans^18–23^.

Thus, defining the contributions of the innate and innate-like cell subsets and their effector cytokines during HIV-1 exposure and early infection in the RM would enhance the overall understanding of rectal HIV transmission and pathogenesis as well as potentially revealing novel avenues for therapeutic interventions. Unfortunately, continual monitoring the RM of individuals at risk of HIV-1 infection to capture the earliest immunological events following exposure is not possible. As an alternative strategy, human rectal explant models have been utilized to study rectal HIV transmission, and particularly to determine the potential efficacy of novel pharmaceuticals to prevent HIV infection^24,25^. In this ex vivo model of HIV-1 transmission, human rectosigmoid biopsies are inoculated with HIV, placed on collagen rafts, and maintained in culture to monitor the accumulation of p24. This model maintains a number of key benefits in approximating HIV transmission and early infection, including (i) utilization of human tissue from a site of exposure, (ii) maintenance of the natural, relevant rectal mucosal architecture^26^, (iii) natural distribution of tissue-resident cellular subsets and their cell-to-cell interactions, and (iv) support of direct infection with HIV-1, even in the absence of immune activating agents typically necessary for infection of peripheral blood cells (e.g. IL-2, PHA, etc.)^24^.

To that end, we sought to define many of the innate and innate-like cell subsets within human RM. We then examined potential relationships between these cellular subsets and HIV-1 replication. Finally, we also interrogated cytokine production within the ex vivo rectal explant model of HIV-1 infection, allowing us to investigate innate, tissue-specific cytokine and effector-function molecules secreted after HIV-1 exposure and during early infection within the RM before the emergence of antigen-specific adaptive immune responses.

## Results

### Quantifying the abundance of innate-like and innate cell subsets within the rectal mucosa

**Figure 1:**
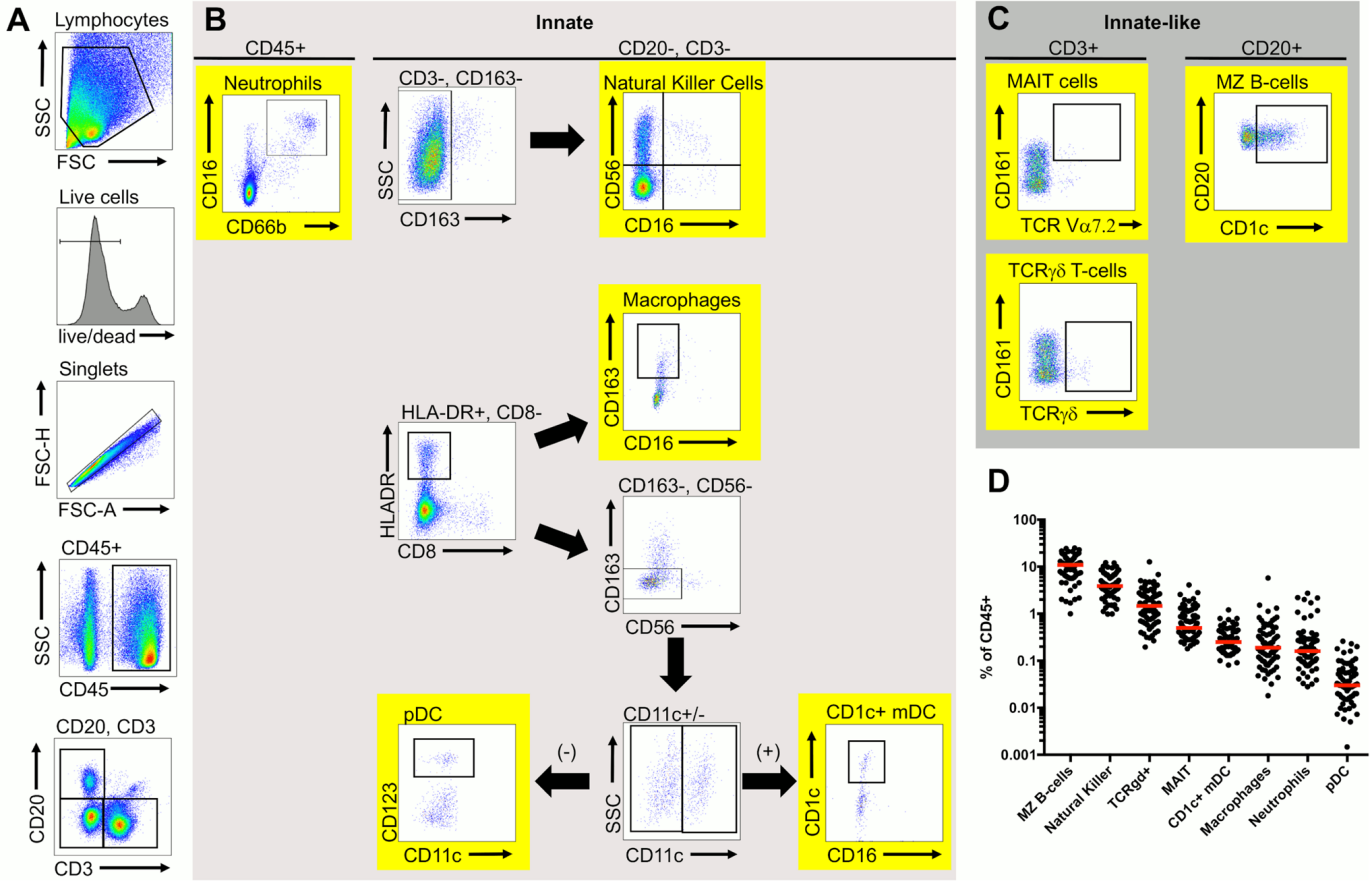
Quantification of innate and innate-like cellular subsets within the rectal mucosa. (**A**) Representative gating strategy for rectal mucosal, (**B**) innate and (**C**) innate-like subsets of CD45 + cells. (**D**) Median (red bar) percentage of innate and innate-like cell subsets as a percentage of CD45 + cells within rectal mucosal biopsies (n = 85 γδ T cells and MAIT, n = 69 all other subsets). pDCs were the least abundant cell subset analyzed (Kruskal–Wallis, p < 0.0001, all comparisons except neutrophils, p = 0.0002). Both MZB (Kruskal–Wallis, p < 0.0001, all comparisons) and NK (Kruskal–Wallis, p < 0.0001, all comparisons, except γδ T cells, p = 0.009) were the most abundant innate cell type quantified.

In order to define the innate and innate-like immune environment within the RM, we first quantified via flow cytometry the following subsets within rectal tissues after collagenase digestion from HIV-negative, sexually transmitted infection (STI)-negative males as a percentage of total CD45 + cells (Fig. 1A–C): neutrophils, macrophages, CD1c + myeloid dendritic cells (mDC), plasmacytoid dendritic cells (pDC), NK, and MZB cells (n = 69), as well as MAIT and γδ T cells (n = 85). In RM, CD123+  plasmacytoid DCs (pDCs) were the least abundant cell subset analyzed (Kruskal–Wallis, p < 0.0001, all comparisons except neutrophils, p = 0.0002) (Fig. 1D). Their presence was rare (median prevalence 0.03% of CD45 + cells, IQR 0.02–0.06%). Conversely, both MZB (Kruskal–Wallis, p < 0.0001, all comparisons) and NK (Kruskal–Wallis, p < 0.0001, all comparisons, except γδ T cells, p = 0.009) were the most abundant cell types quantified with MZB values ranging from 1% to 26.4% of all CD45 + cells (median 11.7%, IQR 6.8–16%) and NK values ranging from 1% to 12.2% (median 3.9%, IQR 2.7–6.5%). γδ T cells were the only additional cell subset regularly present at > 1% of CD45 + cells (median 1.5%, IQR 0.8–2.4%). MAIT cells were found at 0.5% (IQR 0.4–1.0%) median prevalence, and the remaining cell subsets were found at relatively similar abundance in RM: neutrophils (median 0.2%, IQR 0.1–0.3%), macrophages (median 0.2%, IQR 0.1–0.4%), and CD1c + mDC (median 0.3%, IQR 0.2–0.4%).

### Pre-infection MZB and NK cells are associated with HIV replication in the rectal explant model

**Figure 2:**
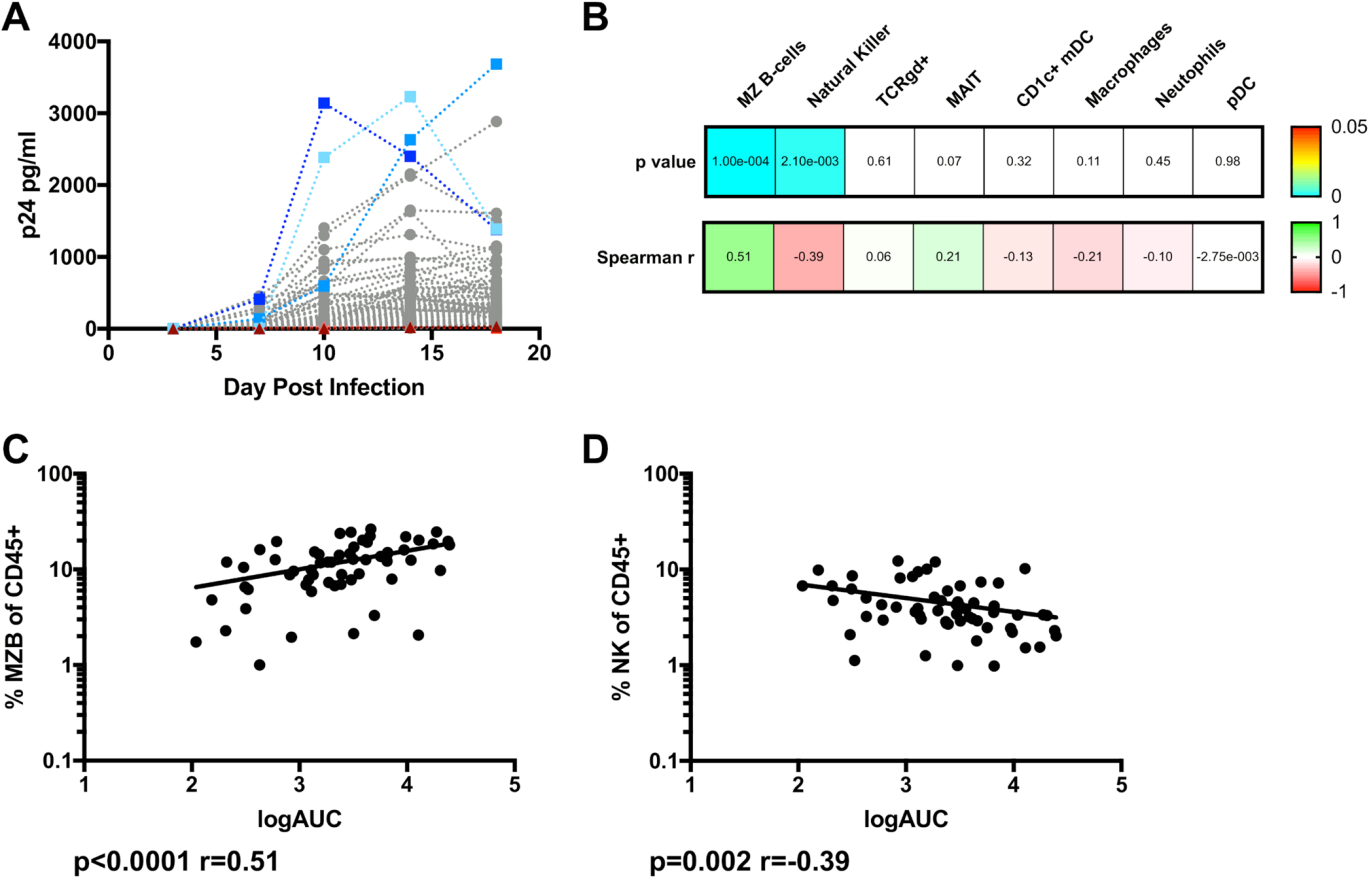
Correlations between NK and MZB cell percentages within the rectal mucosa, and ex vivo HIV-1 replication. Rectal biopsies were exposed to HIV-1 BaL, placed on collagen rafts, and culture supernatants were collected and probed for HIV replication via p24 ELISA over the course of 18 days. (**A**) Longitudinal p24 values for all study participants (n = 86), normalized to biopsy weight. P24 accumulation was above assay limit of detection, but minimal in some individuals (triangles, reds) and substantial in others (squares, blues). (**B**) The median log of the cumulative area under the curve (AUC) of p24 accumulation was calculated and correlated with innate cellular subsets, as a percentage of CD45 + cells. Spearman r and p values indicated. (**C**) A positive association emerged between MZB cells and logAUC p24, (**D**) while NK percentages resulted in a negative correlation with p24.

In parallel with flow cytometric analysis of the RM, three biopsies from the same individuals collected at the same time were each challenged with HIV-1 laboratory variant, BaL, as an ex vivo model of HIV-1 infection of human RM tissue. Supernatants were collected at Day 3, 7, 10, 14, and 18 and assessed for p24 production normalized to biopsy weight. Median p24 concentrations varied substantially between individuals (n = 86, Fig. 2A). To determine whether individual variation in innate or innate-like subsets (Fig. 1) could be contributing to this observed range of HIV-1 replication, Spearman rank correlations were calculated for each cell subset at the baseline vs. the median log Area Under the Curve (AUC) of p24 for each individual. This analysis revealed a positive correlation between the percentage of RM MZB cells and p24 production in parallel RM biopsies (n = 60, p < 0.0001, r = 0.51), and a negative correlation between NK cell abundance and p24 production (n = 60, p = 0.002, r =  – 0.39) (Fig. 2B–D). No other quantified subset correlated with p24 production (Fig. 2B), thus RM MZB and NK were the focus of further characterization.

### MZB and NK cells within rectal mucosal tissues are quantitatively and qualitatively distinct from their circulating counterparts within the peripheral blood compartment

**Figure 3:**
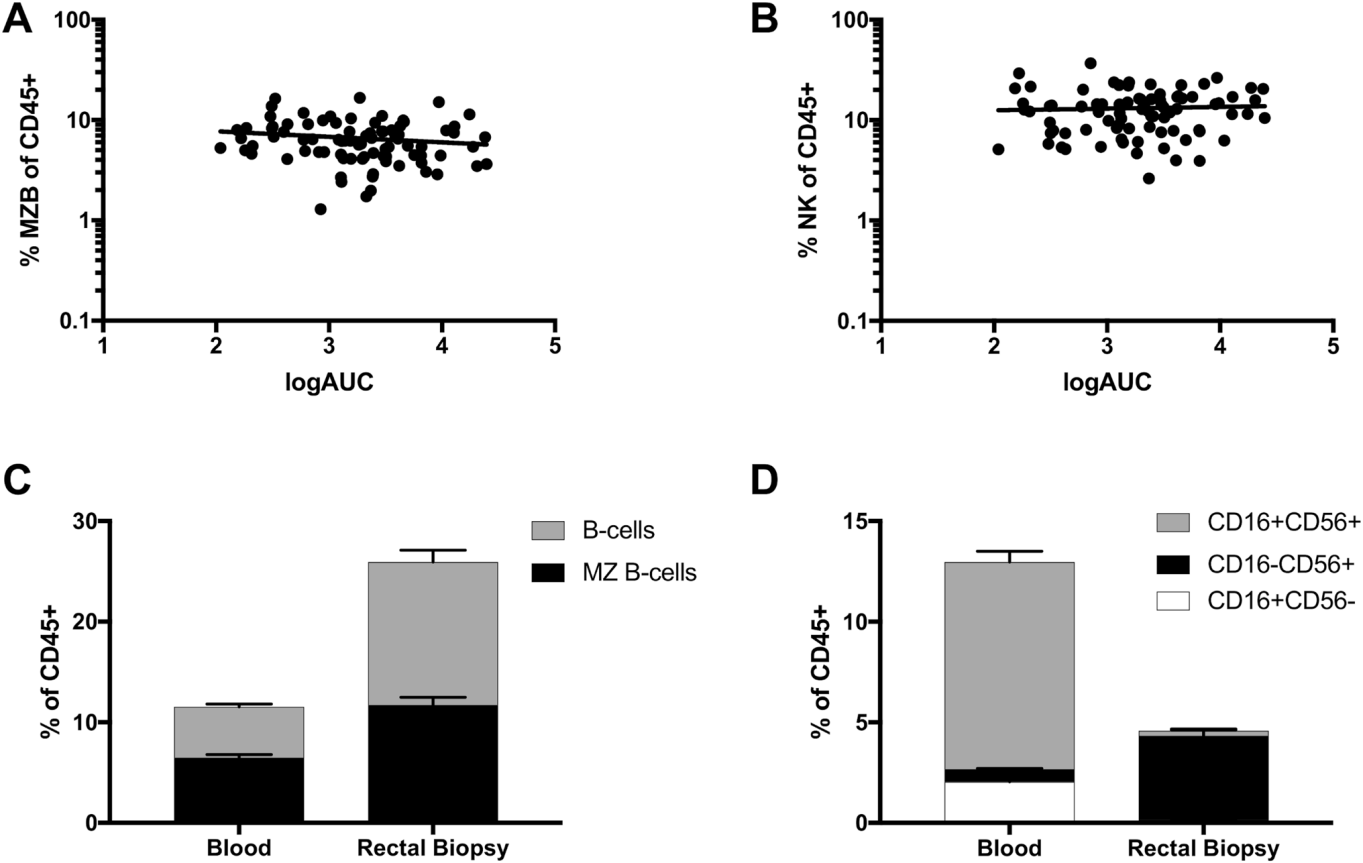
Quantitative differences between RM residing MZB and NK cells, and those circulating in blood. In contrast to the RM residing subsets (Fig. 2), there was no correlation with MZB (**A**) or NK (**B**) percentages within blood and p24 production in the rectal explant. (**C**) Within the RM, there are more B cells, and proportionally more MZB cells, than found within the blood (2way ANOVA, p < 0.0001). (**D**) There are fewer NK within the RM vs. blood, however substantially more demonstrate the CD16-CD56 + phenotype (2way ANOVA, p < 0.0001).

Both MZB cells and NK cells can be found circulating within the human peripheral blood compartment, in addition to their presence in the RM. To compare circulating cells to their RM-residing counterparts, an identical gating strategy (Fig. 1) was utilized with PBMC collected from the same participant at the time rectal biopsies were obtained. In contrast to RM MZB and NK percentages (Fig. 2B–D), no correlation emerged between blood MZB and NK cell subsets and p24 production within parallel rectal explants (Fig. 3A–B). Direct comparison of blood and RM compartments first revealed distinct quantitative differences between both MZB and NK in these subsets. As a percentage of total CD45 + cells, B cells as a whole were more abundant within the RM vs. blood (RM mean = 26%, IQR 14–38%; Blood mean = 12%, IQR 8–14%; two-way ANOVA, p < 0.0001, all comparisons), with MZB cells proportionally more abundant within the RM (RM mean 12%, IQR 7–16%; blood mean 6%, IQR 4–8%; two-way ANOVA, p < 0.0001) (Fig. 3C). Compartmental differences were also seen within the NK population (Fig. 3D). NKs were more abundant within the blood vs. the RM (RM mean 5%, IQR 1–6%; Blood mean 13%, IQR 8–16%; two-way ANOVA, p < 0.0001, all comparisons), and cell surface markers were highly divergent and differentially distributed between CD56 + , CD16 + , and dual-positive NK between the two compartments. This differential was not due to differences in processing protocols between blood and RM cells, as blood cells carried through the RM processing protocol (with Collagenase IV and DNase, see Methods) were identical to unprocessed cells (dns), and tissue-specific variation in CD16 expression on NK cells is expected^27^.

**Figure 4:**
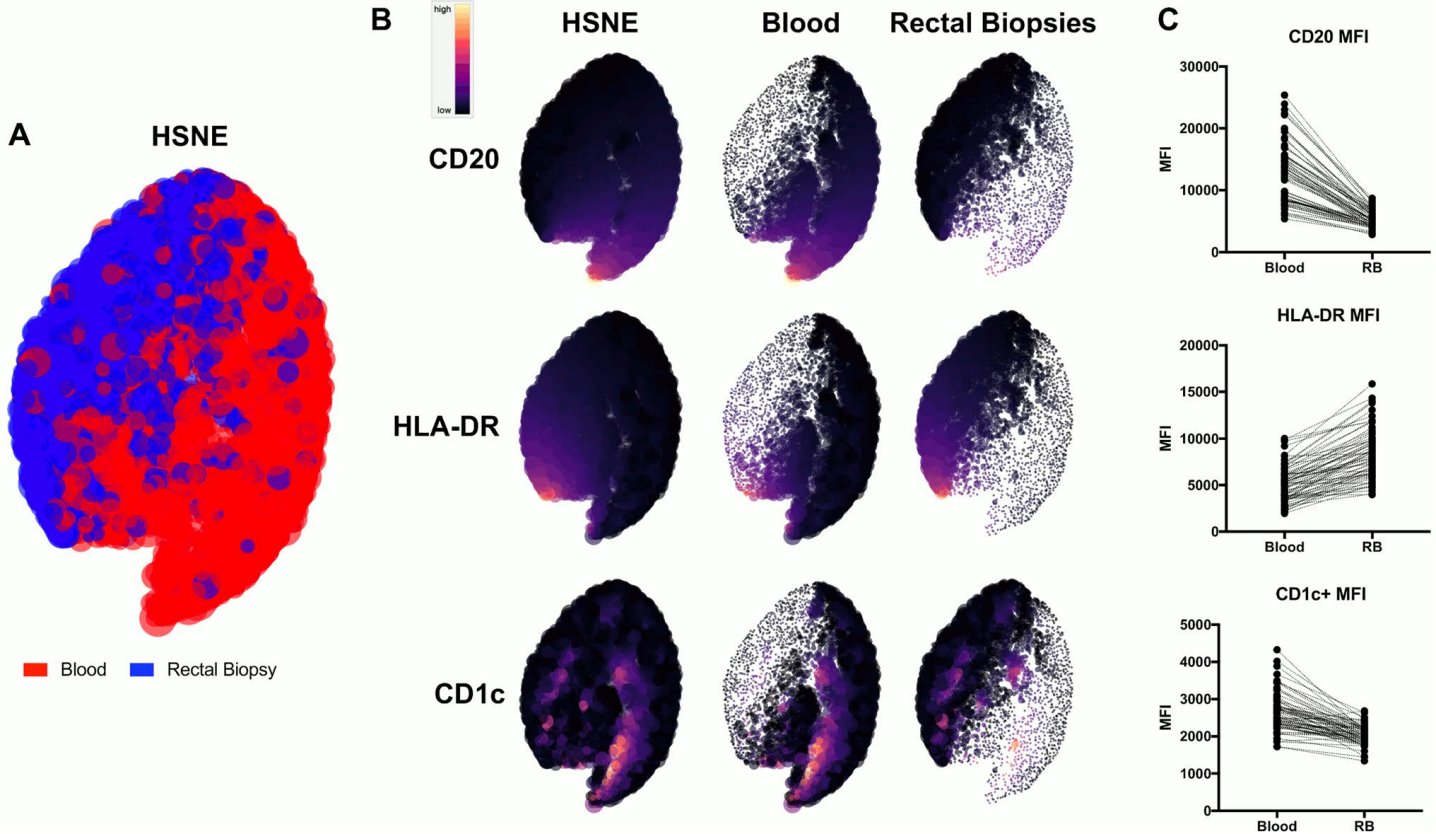
HSNE analysis of blood and RM residing MZB cells. Analysis was performed with matched blood and RM MZB cells, defined as CD45 + , CD3−, CD20 + , HLA-DR + , CD1c + (no more than 5000 events/participant/compartment). (**A**) HSNE analysis illustrated a polarization of blood (red) and RM (blue) MZB cells. (**B**) Heatmap analysis illustrates higher CD20 and CD1c expression on MZB from blood, while increased HLA-DR expression on RM residing MZB. **C**) MFI comparisons on the same cells demonstrate statistical differences in expression of these markers on blood vs. RM MZB (Wilcoxon test, p < 0.0001, all markers).

**Figure 5:**
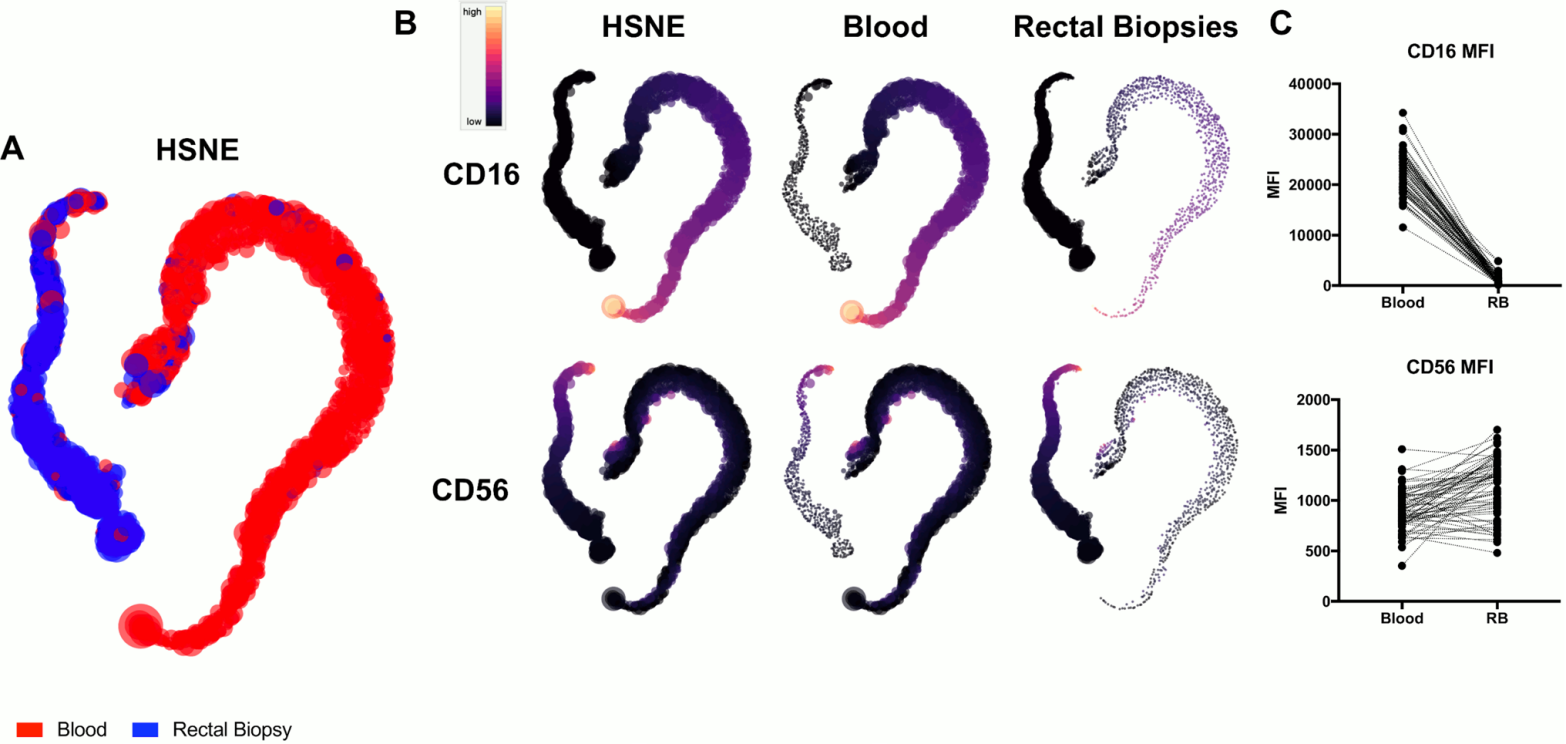
HSNE analysis of blood and RM residing NK cells. Matched blood and RM NK cells were compared in this instance, defined as CD45 + , Lin− , CD16± , CD56± (no more than 5000 events/participant/compartment). (**A**) This HSNE analysis resulted in discrete populations of blood (red) and RM (blue) NK cells. (**B**) Heatmap analysis illustrates a near absence of CD16 on RM residing MZB. **C**) MFI comparisons on the same cells demonstrate statistical differences in expression of both CD16 and CD56 on blood vs. RM MZB (Wilcoxon test, p < 0.0001, all markers).

To gain further insight into these differences, Hierarchical Stochastic Neighbor Embedding (HSNE) analysis was performed on MZB and NK cells^28^. To prevent a single participant or tissue compartment from dominating or skewing results, for MZB cells, no more than 5000 CD45 + , CD3-, CD20 + , HLA-DR + , CD1c + events from each participant from each compartment were analyzed based on CD20, HLA-DR, and CD1c expression. Based on these markers, blood and RM MZB cells did not segregate into discrete populations (Fig. 4A). However, clustering of cells was polarized based on their origin. While all analyzed cells were positive for CD20, HLA-DR, and CD1c, heatmap analysis illustrated blood MZB clustered based on elevated CD20 and CD1c expression, while RM MZB expressed relatively higher levels of HLA-DR (Fig. 4B). Mean fluorescence intensity (MFI) from matched blood and RM-derived MZB also revealed differential expression of these three markers between blood and RM-residing MZB (Fig. 4C, Wilcoxon test, p < 0.0001, all markers). Similar analysis of blood and RM-residing NK cells was performed on CD45+ , Lineage-, CD16± , CD56 ± . In this analysis, two distinct populations emerged (Fig. 5A), based substantially on the near absence of CD16 on RM-residing NK (Fig. 5B). MFI from matched blood and RM-residing NK cells again revealed statistical differences in expression of both CD16 and CD56 within NKs from these two compartments (Fig. 5C, Wilcoxon test, p < 0.0001, all markers).

### Diverse innate anti-viral cytokines and effector molecules are secreted in the ex vivo rectal explant challenge model and correlate with productive HIV-1 infection

The ex vivo rectal explant model of HIV-1 infection provides a unique opportunity to explore a potentially tissue-specific cytokine response to HIV in human RM in the days following HIV exposure, before the adaptive immune system evolves antigen-specific responses. To begin to elucidate the innate and innate-like cytokine environment generated in this model in the earliest days post-HIV infection, we quantified the presence of 22 molecules longitudinally, from Day 3 to Day 14 post infection, in a subset of productively infected explants (n = 26, p24 logAUC range 2.2–4.4) (Supplemental Figs. 1, 1). Mean and median longitudinal concentrations were at or below the assay limit of detection for eight molecules: IL-2, IL-4, TNFα, IFN-λ1, IL-12p70, IFN-α2, IFN-λ2/3, and IFN-β2. Concentrations of these cytokines were similarly low/undetectable in the mock infected biopsy (biopsies exposed to culture media rather than viral challenge media), suggesting they are not present at appreciable concentrations in the rectal explant model; however, it is possible that they were present at levels below the detection threshold, or were expressed and degraded prior to the Day 3 sampling time-point.

The remaining 14 molecules were present within the supernatants of infected rectal explants: IL-1β, IL-6, IL-8, IL-10, IL-17A, IFN-γ, IP-10, GM-CSF, sFas, sFasL, Granzyme A (GZA), Granzyme B (GZB), Granulysin, and Perforin (Supplemental Figs. 1, 1). As expected, no assayed cytokine or effector molecule was detectable in appreciable concentrations within the culture media used to maintain the biopsies (Supplemental Figs. 1, 1). Several molecules were present at high concentrations within the viral challenge stock media (> 10 pg/ml, including IFN-γ, GZA, GZB, GM-CSF, IL-2), likely due to propagation of the virus in human PBMC (Supplemental Figs. 1, 1). However, the complete absence of IL-2 in all infected biopsies (despite > 250 pg/ml concentration in the viral stock), along with the longitudinal kinetics of IFN-γ, GZA, GZB, GM-CSF in the infected biopsies (including complete absence in some biopsies) (Supplemental Figs. 1, 1) suggests post-challenge washing of biopsies was sufficient to remove excess viral challenge media, and the detected concentrations of these molecules in the infected biopsies represent de novo production, rather than carry-over from the challenge stock.

**Figure 6:**
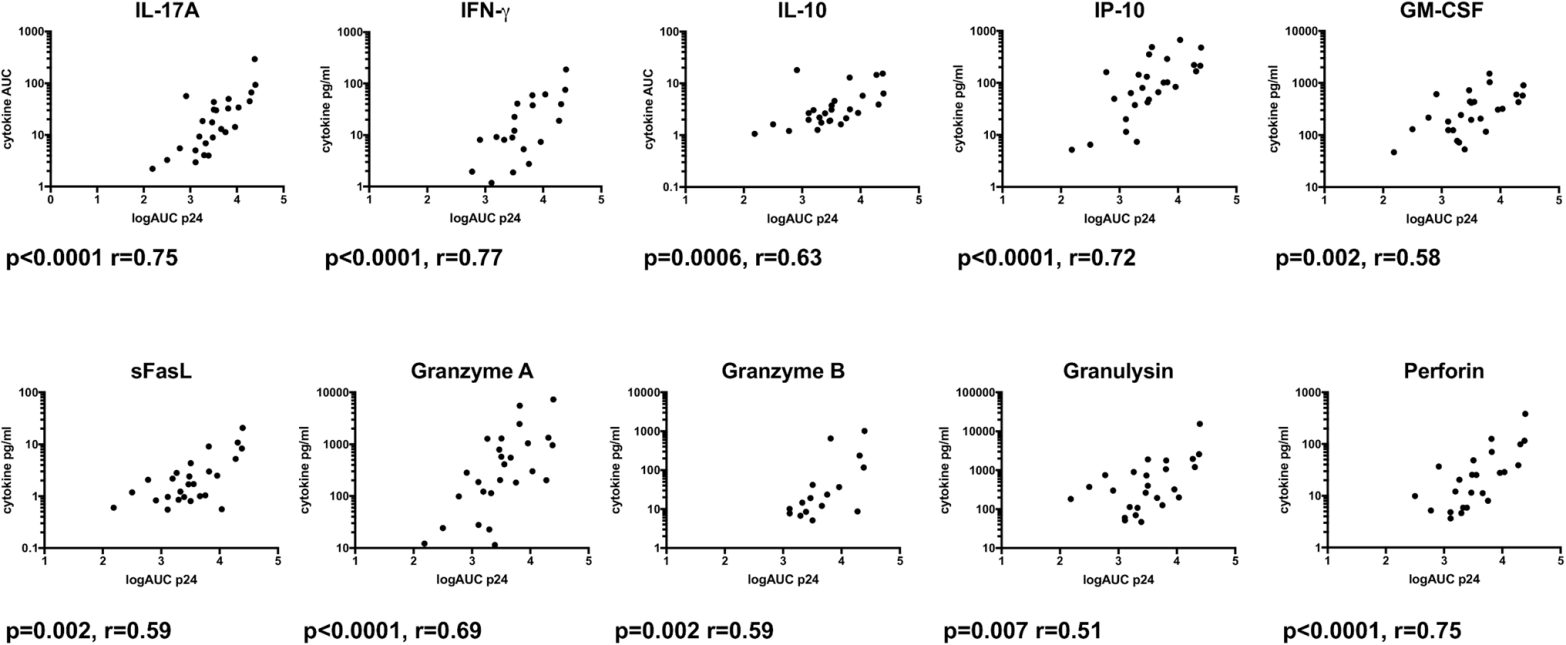
Associations between cytokine and effector molecule production and p24 production within the rectal explant model of infection. Cytokine and effector molecule production was quantified, longitudinally, in a subset of rectal explant supernatants (n = 26). Spearman analysis revealed correlations between log p24 AUC (x axis) and IL-17A, IFN-γ, IL-10, IP-10, GM-CSF, sFasL, GZA, GZB, Granulysin, and Perforin AUC (y axis). Spearman p and r values indicated. Data points where molecules were not detectable (zero values) are not visible on graph due to log scale, however all values were included in correlation analysis.

To determine the relationship between the 14 detectable molecules and HIV-1 replication, longitudinal soluble molecule concentrations were normalized to biopsy weight. The resulting AUC (from days 3–14) was calculated for each molecule, and correlated with the corresponding normalized p24 logAUC values (Spearman, Fig. 6). Significant correlations (controlled for multiple comparisons, threshold of significance reduced to p < 0.0076 by two-stage linear step-up procedure of Benjamini, Krieger and Yekutieli) were observed with IL-17A (r = 0.75, p < 0.0001), IFN-γ (r = 0.77, p < 0.0001), IL-10 (r = 0.63, p = 0.0006), IP-10 (r = 0.72, p < 0.0001), GM-CSF (r = 0.58, p = 0.002), sFasL (r = 0.59, p = 0.002), GZA (r = 0.69, p < 0.0001), GZB (r = 0.59, p = 0.002), Granulysin (r = 0.51, p = 0.007), and Perforin (r = 0.75, p < 0.0001). IL-6, IL-8, sFas, and IL-1β did not correlate with p24 production. Of note, IL-6, IL-8, IL-1β, and GM-CSF were also elevated in the mock infected biopsy, thus are possibly secreted in response to the injury induced by the biopsy procedure and cell death, and potentially obfuscated our ability to fully elucidate the relationship between these inflammatory cytokines and HIV replication in this model.

## Discussion

The findings from this study demonstrate that innate immune cell subsets, as a whole, are a minority population of CD45 + cells within the RM, yet some are potentially influencing HIV production within the RM. Most of the subsets defined here were present at or below 1% of total CD45 + cells. A notable exception to this trend were the MZB cells, found at a median of > 10% of CD45 + cells. MZB cells are emerging as a cell type of interest in HIV-1 transmission and pathogenesis. Recent studies have reported decreased numbers of MZB in the blood and within the female reproductive tract of HIV-exposed but uninfected commercial sex workers^22,23^. This suggests that MZB cells could be contributing to HIV transmission within the female genital tract, although mucosal B cells at this site are relatively rare^29^. Though MZB have not been specifically quantified in the male reproductive tract, CD19 + B cells as a whole comprise 1–3% of CD45 + cells^30^. This is in contrast to the MZB distribution we observed within the RM, where we noted a substantial proportion of CD45 + cells are MZB cells. Also, a recent study by Liechti et al.noted an inverse correlation between MZB within the blood and CD4 + T cell counts, and a positive correlation between MZB and viral load in the setting of chronic HIV-1 infection^31^. Congruent with these previous findings, we also observed a positive correlation between MZB and p24 production after ex vivo challenge of rectal tissue with HIV.

The precise mechanisms by which MZB could contribute to HIV transmission and replication has yet to be elucidated. MZB may be directly involved in HIV-1 replication via *trans* infection of tissue-resident CD4 + T cells. Although macrophages and DCs are more commonly associated with *trans* infection, there are higher proportions of MZB than macrophages, mDC, or pDC in RM, and it has been shown previously that peripheral B cells mediate *trans* infection as efficiently as other APC subsets^14^. Although traditional HIV-binding receptors such as CD21 and DC-SIGN were not quantified here, studies have reported MZB cells are capable of binding directly to HIV gp120^23^. When combined with unique intrinsic features of the rectal mucosa ex vivo HIV challenge model (i.e. the absence of requirement for immune activating agents commonly necessary for infection of PBMC), it is possible that the three-dimensional structure of the RM is maintaining cell-to-cell contacts thus facilitating *trans* infection. It is also feasible that within the RM, MZB are part of an immunological ecosystem that promotes increased HIV susceptibility of target cells. Low levels of CD4 + T cell activation have been associated with various HIV-exposed but uninfected populations^32^. In the context of murine studies, where MZB are generally only found within follicles, these cells are potent activators of CD4 + T cells^33^. Thus, MZB could indirectly contribute to p24 accumulation by robustly activating local CD4 + T cells within RM tissues. Mechanistically defining the relationship between MZB cells and HIV replication could prove critical for better understanding RM transmission and in the development of novel biomedical HIV prevention interventions. This will necessitate further utilization of the human rectal explant model, as our data demonstrate that human MZB circulating in peripheral blood are quantifiably different from those residing in the RM, even in comparative analysis restricted to a modest group of surface receptor markers. However, prior to this, it will be beneficial to utilize exploratory approaches, such as single-cell RNAseq, to characterize these MZB-like cells residing within non-splenic tissue compartments of humans. Ongoing studies within the field would allow for direct comparison and quantification of transcriptomic similarities of these RM-residing B cells to traditional splenic MZB, as well as circulating MZB and other B cell subsets^34^. This will provide critical foundational information as to the natural function of these cells within the RM, as well as providing insight into how these cells could potentially participate in propagating HIV replication. In addition, use of the human explant model is necessitated by limitations in animal models considering that circulating and tissue-residing MZB are not found in mouse models and may be absent in NHP models as well^35^.

Of the potential innate negative regulators of HIV replication within the RM, we identified a negative correlation between NK percentages and p24 accumulation. That is, biopsies from participants with reduced p24 production also displayed a higher proportion of NK cells within the RM of parallel biopsies. In this study, we considered lineage negative, CD163− (RB) or CD14− (blood), CD56 + and/or CD16 + cells as a pooled population, and did not stratify NK into the archetypical categories of CD56^bright^ and CD16^+^CD56^dim^, due to the diversity of expression of these molecules on NK residing in numerous tissues vs. traditional NK populations within peripheral blood^36^. In concordance with studies characterizing NK residing in other tissues, we also observed that this population of cells within the RM does not express high levels of CD16. This is in contrast to approximately 40–60% NK cells that reside within the penile mucosal tissue, which do appear to express CD16^30^. And, within the female reproductive tract, CD16 is expressed by NK residing within the ectocervix, while uterine NK are notable for the absence of CD16^37,38^. In this study, utilizing RM tissue, the absence of both CD16 and ADCC-mediating antibodies (all participants were HIV seronegative), the potential mechanism of action by which RM NK could reduce HIV replication would likely be mediated via any number of NCR, KLR, and KIR, each of which can have a profound impact on NK-mediated killing of HIV infected cells^8,9^. For example, HIV has evolved mechanisms to reduce the presence of Class I Major Histocompatibility Complexes on the surface of infected cells to evade antigen-specific T-cell responses (via HIV proteins Vpu and Nef), however this renders infected cells more susceptible to recognition and elimination by NK. The absence of self-ligands on the surface of infected cells (i.e. ‘missing self’) could lead to activation of NK-killing, due to the absence of inhibitory signals mediated by KLR NKG2A or inhibitory KIR. While we have not identified the exact cellular source of the cell-killing effector molecules detected in this study (sFasL, GZA, GZB, granulysin, and perforin), it is possible that NK responses are contributing to the production of these molecules. Furthermore, this conclusion would suggest that just as HIV-1 out-maneuvers the B cell and T cell responses, HIV is likewise capable of outpacing the relatively rapid NK response within the RM^39^, similar to what has been observed within the female genital tract of SIV-infected rhesus macaques^40^. Our findings do introduce the possibility that increasing the presence of NK within the RM, or optimizing the anti-viral capabilities of RM-residing NKs, could contribute to an immunological environment that is not conducive to HIV replication. Similarly, as both RM-NK and uterine NK lack expression of CD16, similar enhancement of ADCC-independent cytotoxic pathways of NK residing in both of these tissue compartments could provide additional protection against HIV infection. Because RM-resident NKs are distinct from those found in PBMC, detailed phenotypic and genetic characterization of these RM-residing NK cells will be necessary to elucidate the inhibitory capabilities of this subset against HIV infection. This avenue of research must be expanded to explore potentially distinctive features of additional subject-derived HIV isolates (T/F and non-T/F) of numerous subtypes, to mechanistically define the selective pressures exerted by NK and other innate and innate-like subsets^41^.

In addition to identifying associations between RM-residing MZB and NK cells and HIV-1 replication, the explant model utilized here allowed for the identification of associations between p24 production and a number of assayed cytokines and effector molecules potentially released early after HIV infection, before the adaptive immune response matures. In the absence of this RM-specific data, elevated cytokines have been described in the context of systemic levels of inflammatory and anti-viral cytokines present in blood during acute/early HIV-1 infection^42,43^. These systemic studies provide an informative point-of-comparison for our observations, and we note a number of cytokines typically elevated systemically are also associated with p24 production in the rectal explant model. For example, IFN-γ was one of the first cytokines identified as elevated in serum during acute HIV-1 infection^42,43^. IFN-γ is also upregulated within the RM during chronic HIV infection, persisting even after initiation of ART^44^. We also identified IFN-γ as critical to propagation of HIV-1 infection within the rectal mucosa. Additional concordant cytokines previously reported to elevated in blood in acute HIV infection and now seen in our study within the rectal explant model include IP-10, IL-10, and GM-CSF, reemphasizing their role in the pathogenesis of early HIV infection^45^.

Unique to the rectal explant model, however, was the identification of the strong association of IL-17A production with p24 accumulation. Though it has been conceptually hypothesized that IL-17 could influence HIV replication (reviewed in^46^), this relationship has been difficult to elucidate in humans. Serum levels of IL-17A are not elevated in most individuals during acute infection^47^, nor in NHP models utilizing pathogenic and non-pathogenic SIV variants^48^. It is logical that this relationship is observable directly in ex vivo challenged rectal explants, even though it does not emerge in systemic studies of acute HIV infection, as there are tissue-dependent roles for IL-17A in both healthy and disease states^49^. Within the gut mucosa, IL-17A plays a vital role in balancing interactions with the microbiome and in maintaining intestinal mucosal integrity. Overexpression of IL-17A is associated with inflammatory bowel disease, while attempts to suppress IL-17A production in these instances can lead to further intestinal epithelial injury and colitis^49^. While our observations have identified IL-17A as a facilitator of HIV replication and, as such, it could be an attractive novel target for prophylactic or therapeutic interventions, the importance of maintaining homeostatic levels of IL-17A in human RM may deem it a poor target for use in clinical interventions. Future studies should capitalize on the rectal explant model to determine whether targeting IL-17A production in a localized, temporary manner in RM tissues during HIV-1 exposure limits, or promotes, HIV transmission.

Also notable in the explant model utilized here are the cytokines elevated upon systemic HIV infection, such as Type 1 interferon, that were not elevated in the RM explant model^47^. Neither IFN-α nor IFN-β were present at substantial concentrations in supernatants at any point between Day 3 and Day 14 post infection. It is hypothesized that these cytokines could act as a strong selective pressure on transmitted/founder viruses, restricting the replication of the most susceptible genetic variants, contributing to the genetic bottleneck frequently observed during transmission^50^. Notably, in female rhesus macaques, pDCs within the vaginal mucosa are strongly positive for both IFN-α and -β^51^. It is possible that the absence of these cytokines in the rectal explant model are due to the dearth of pDCs in the RM and the inability to recruit additional cells to the site of infection in the explant model. Alternatively, the absence of substantial IFN-α in this study might be attributable to sex-specific differences in pDC function^52^. Finally, it is also possible that the laboratory HIV-1 variant, BaL, does not have the same capacity to induce Type 1 interferon as patient-derived isolates^53,54^. Again, further utilization of the rectal explant model with subject-derived T/F and non-T/F viruses will be critical for quantifying differences in i) cytokine induction and ii) susceptibility to the anti-viral cytokines we observed here, to further define the cytokines exerting selective pressure on the virus at the time of rectal transmission.

Limitations of the current study include a single-sex cohort, and use of a single HIV laboratory variant; follow-up studies are being planned in our lab to address these limitations. The rectal explant challenges were also carried out with the use of cell-free viral stocks. Exposure of RM tissue to HIV infected cells and cell-associated virus found within the complex milieu of semen might result in different selective pressures exerted by different immune cells and cytokines within the RM, and should be explored in future studies as well^55,56^. Furthermore, it will be critical to include additional markers relevant to the identified cellular subsets of interest in future studies to fully elucidate potential mechanisms of action for our observed associations with HIV replication (e.g. quantification of CD21, DC-SIGN, IgM for MZB; KIRs, NCRs, KLRs for NK). Additionally, all participants included in this analysis were negative for bacterial STIs. It is possible that RM immuno-environments that include symptomatic or asymptomatic STI infections, or an immunological landscape created by any number of inflammatory bowel conditions, are radically different from the distribution of immune cell subsets observed in this study. In alternative ‘inflammatory’ environments, it is possible additional immune cell subsets and inflammatory cytokines are associated with HIV replication in the ex vivo model.

The present study is also limited in that the observations are currently correlative. However, our findings are leading to focused, hypothesis-driven mechanistic studies within the explant rectal challenge system in future studies. For example, flow cytometry panels focused on defining features of MZB and NK cells can be utilized to interrogate biopsies over the course of ex vivo HIV infection. We anticipate NK cells might proliferate in response to HIV infection of RM tissue, while the expression of activation markers, such as HLA-DR and CD38, might increase on both MZB and NK cell subsets. Flow analysis after HIV infection would also allow for intracellular staining to identify the cellular sources of the cytokines and effector molecules identified in the explant supernantants in the current study. Furthermore, if NK cells within the RM are targeting the first HIV infected cells within this tissue in an ADCC-independent mechanism, this activity could be augmented in future studies by supplementing the explant supernatant with NK activators, such as IL-15, or check-point inhibiting antibodies that could enhance ADCC-independent killing pathways, such as αNKG2a or αKIR antibodies. It also might be possible to interrogate the role of MZB via antibodies without disrupting the three-dimensional structure of the RM biopsies via inhibition of potential B-cell mediators of *trans* infection, such as αCD21 or αDC-SIGN. Furthermore, within the current analysis, we cannot determine whether the positive association between cytokines and p24 is due to i) cytokine production emerging as a direct result of p24 production and inflammation, or, ii) the presence of these cytokines is further augmenting and enhancing HIV replication. In future analyses, explants can be pre-treated with exogenous cytokines or cytokine neutralizing antibodies to directly elucidate cause-and-effect relationships between the associations identified here.

In summary, we have identified a number of unique innate immune cell subsets and cytokines associated with the replication of HIV-1 in human rectal mucosal tissues after viral exposure. These observations would not have been possible without this novel use of human RM and the rectal explant model of HIV infection, due to the tissue-specific and potentially species-specific features of MZB cells, NK cells, and IL-17A. Future studies will likewise necessitate the use of human RM tissue to further characterize these cellular subsets, to identify the source of IL-17A, and to determine the mechanisms by which they are contributing to or restricting HIV replication in RM tissues. This further investigation could prove essential in pursuit of a better understanding of the process of HIV-1 transmission across RM tissue and also in developing strategic methods for reducing RM transmission events.

## Methods

### Study population

This study obtained approval from Emory University Institutional Review Board (IRB). All procedures and experiments were performed in accordance with appropriate guidelines and regulations. Written informed consent was obtained from all participants. Participants were healthy, HIV-negative, aged 18–69 years (median age 36), from the Atlanta metropolitan area, and tested negative for rectal gonorrhea, rectal chlamydia, and syphilis at the time of sampling. Participants included in this analysis were recruited for a larger parent study focused on men who have sex with men that enrolled men who do and do not regularly engage in receptive anal intercourse; therefore, women were not eligible for enrollment. Individuals who were determined to be high risk for complications from rectal biopsy procedures or who intended to take pre-exposure prophylaxis medications during the study were not enrolled. Biopsies were collected 3 to 10 cm from the anal verge via rigid sigmoidoscopy with no prior bowel preparation. During the same visit, PBMC were collected via BD Vacutainer Cell Preparation (CPT) tubes.

### Blood and rectal mucosal cell phenotyping

Blood lymphocytes were collected via centrifugation of CPT tubes, while mononuclear cells from the RM were isolated as described previously^57^. In brief, 5 rectal biopsies were mechanically (scalpel, blunt-end syringe) and chemically (200 U/ml Collagenase IV, 2 U/ml DNase, 37° C 2 h) processed to release mononuclear cells present within the RM. After red blood cell lysis (#118–156-101) and filtration (40 um), cells were stained with the following antibodies: Live/Dead Aqua (Thermo Fisher #L34957), CD45 (3 μl, Clone 2D1, Biolegend #368,516), CD3 (3 μl, Clone UCHT1, Biolegend #300,424), CD8 (3 μl, Clone SK1, Biolegend #344,712), HLADR (3 μl, Clone L243, Biolegend #307,636), CD16 (3 μl, Clone 3G8, Biolegend 302,040), CD56 (3 μl, Clone 5.1H11, Biolegend #362,550), CD66b (3 μl, Clone G10F5, Biolegend #305,108), CD11c (5 μl, Clone 3.9, Biolegend #301,638), CD123 (3 μl, Clone 6H6, Biolegend #306,012), CD1c (3 μl, Clone L161, Biolegend #331,532), CD86 (3 μl, Clone IT2.2, Biolegend #305,440), CD14 (blood cells only) (5 μl, Clone M5E2, Biolegend #301,806), CD163 (RM cells only) (5 μl, Clone GHI/61, Biolegend #333,606), CD161 (3 μl, Clone HP-3G10, Biolegend #339,918), TCR Vα7.2 (3 μl, Clone 3C10, Biolegend #351,720), TCRγδ (3 μl, Clone B1, Biolegend #331,226). Staining was performed with Brilliant Stain Buffer (BD Horizon #566,385), True-Nuclear Transcription Factor buffers (Biolegend #424,401) or Cytofix/Cytoperm (BD 51-2090K2), in accordance with manufacturer’s instructions. Samples were collected on a BD Fortessa, with file analysis within FlowJo 10.5.3. Cells were defined in the following manner: Live, singlets, lymphocytes, CD45 +: Neutrophils: CD66b + , CD16 + ; NK: CD20-, CD3−, CD163−, CD56 + and or CD16 + ; Macrophage: CD20−, CD3−, CD8−, HLA-DR + , CD163 + ; pDC: CD20−, CD3−, CD8−, HLA-DR + , CD56−, CD163−, CD11c−, CD123 + ; mDC: CD20−, CD3−, CD8−, HLA-DR + , CD56−, CD163−, CD11c + , CD16−, CD1c + ; MZB: CD3−, CD20 + , HLA-DR + , CD1c + ; MAIT: CD3 + , CD161 + , TCRvα7.2 + ; γδ T cell: CD3 + , TCR γδ + .

### Ex vivo explant challenge

Three biopsies from each participant were individually weighed, and exposed to HIV-1 BaL (10^2.8^ TCID_50_ in a volume of 250 μl media) for 2 h (37 °C, 5% CO_2_) in a 24-well plate. After viral exposure, each biopsy was extensively, serial washed in sterile PBS (5 × 500 μl), and placed on a pre-soaked, pre-warmed collagen raft (Pfizer #00,300,090,315,085) in 1 ml complete media (RPMI 1640 with 10% FBS, Gentamicin Sulfate, and Zosyn). At set post-infection timepoints (Day 3, 7, 10, 14, 18), 700 μl media was removed, and replaced with 700 μl complete media. Collected supernatants were stored in -30° C until p24 or cytokine analysis. P24 was quantified via ELISA (ABL, Inc. #5447) according to manufacturer’s instructions. P24 values were normalized to biopsy weight (median weight 8.1 mg, IQR 6.8–10 mg).

Cytokines and effector molecules were quantified utilizing BioLegend LEGENDplex CD8/NK Panel (BioLegend, #740,267) and Human Anti-Virus Response Panel (BioLegend, # 740,390). Samples were diluted 1:2 in assay buffer and run according to manufacturer’s protocols in a V-bottom plate. Events were collected on a BD Fortessa, with file analysis performed with BioLegend’s proprietary LEGENDplex Data Analysis Software. Cytokines below the standard range calculated for each assay/cytokine were normalized to zero. Final cytokine concentrations utilized for associations with p24 production were normalized to biopsy weight. Four cytokines were assayed in both kits: IL-6, TNFα, IFNγ, and IL-10. Results from CD8/NK kit are reported here. Low/undetectable concentrations of TNFα lead to sporadic detection of this cytokine, and resulted in no correlation between AUC for this cytokine between kits (Spearman, p = 0.38), and neither correlated with p24 AUC. However detectable cytokines were highly correlated between kits (IL-6, p = 1.7e-10; IFNγ, p = 6.7e-11; IL-10, p = 1.97e-7), with similar correlations to p24 AUC (Antiviral kit: IL-6, ns; IFNγ, r = 0.80, p < 0.0001; IL-10, r = 0.53, p = 0.006) thus this data is only presented once.

### Statistical analyses

All graphing, calculations, and analyses were performed in Prism 7.0. Kruskal–Wallis multiple comparisons analysis was used to evaluate frequencies of each innate cell type within the RM. Spearman correlations were utilized for associations between p24 logAUC and cell subsets, and cytokine AUC. For cytokine and effector molecule correlations, the threshold of significance was adjusted to p < 0.0076, after correction for multiple comparisons (Benjamini, Krieger and Yekutieli). Two-way ANOVA was used to compare B cells and NK composition of the blood vs. RM. Wilcoxon test was used to compare MFI of markers between matched blood and RM subsets. HSNE diagrams were generated with Cytosplore^28^. We also conducted sensitivity analyses to examine the influence of sexual behavior on our results. Of note, there was no statistical difference between HIV replication (Mann–Whitney, p > 0.05), MZB (p > 0.05), or NK (p > 0.05) when data were stratified between men who reported receptive anal intercourse and men who did not. Therefore, data for the combined total cohort are reported in this manuscript.

## Supplementary information

Supplementary Legend.

Supplementary Figure S1.

Supplementary Figure S2.
