# Supplementary material for: Ex vivo rectal explant model reveals potential opposing roles of Natural Killer cells and Marginal Zone-like B cells in HIV-1 infection
Source: Sci Rep. 2020 Nov 19;10:20154. doi: 10.1038/s41598-020-76976-5 (PMC7677325; doi:10.1038/s41598-020-76976-5)
Supplement: Supplementary file 1 — Supplementary Legend. [file 41598_2020_76976_MOESM1_ESM.docx]

**Supplemental Figure 1. Quantification of CD8/NK cytokines and effector molecules present within explant challenge supernatant.** Raw longitudinal concentrations for each participant (n=26, gray) is presented here (x axis days post-infection, y axis raw molecule concentration) with the overall mean (red) and median (black). Mean values obtained from biopsies cultured in media alone are presented in blue. Values calculated from culture media (green) and viral challenge stock (orange) are noted at Day 1. Black dashed line represents assay limit of detection for each cytokine. Data points where molecules were not detectable (zero values) are not visible on graph due to log scale.

**Supplemental Figure 2. Quantification of Antiviral cytokines present within explant challenge supernatant.** Raw longitudinal concentrations for each participant (n=26, gray) is presented here (x axis days post-infection, y axis raw molecule concentration) with the overall mean (red) and median (black). Mean values obtained from biopsies cultured in media alone are presented in blue. Values calculated from culture media (green) and viral challenge stock (orange) are noted at Day 1. Black dashed line represents assay limit of detection for each cytokine. Data points where molecules were not detectable (zero values) are not visible on graph due to log scale.
