# Supplementary material for: Ex vivo rectal explant model reveals potential opposing roles of Natural Killer cells and Marginal Zone-like B cells in HIV-1 infection
Source: Sci Rep. 2020 Nov 19;10:20154. doi: 10.1038/s41598-020-76976-5 (PMC7677325; doi:10.1038/s41598-020-76976-5)

Ex vivo rectal explant challenge model reveals potential opposing roles of Natural Killer cells and Marginal Zone-like B cells in HIV-1 infection

S. Abigail Smith, Phillip M. Murray, Praveen Kumar Amancha, Cassie G. Ackerley, Yi-Juan Hu, Rama R. Amara, Colleen F. Kelley

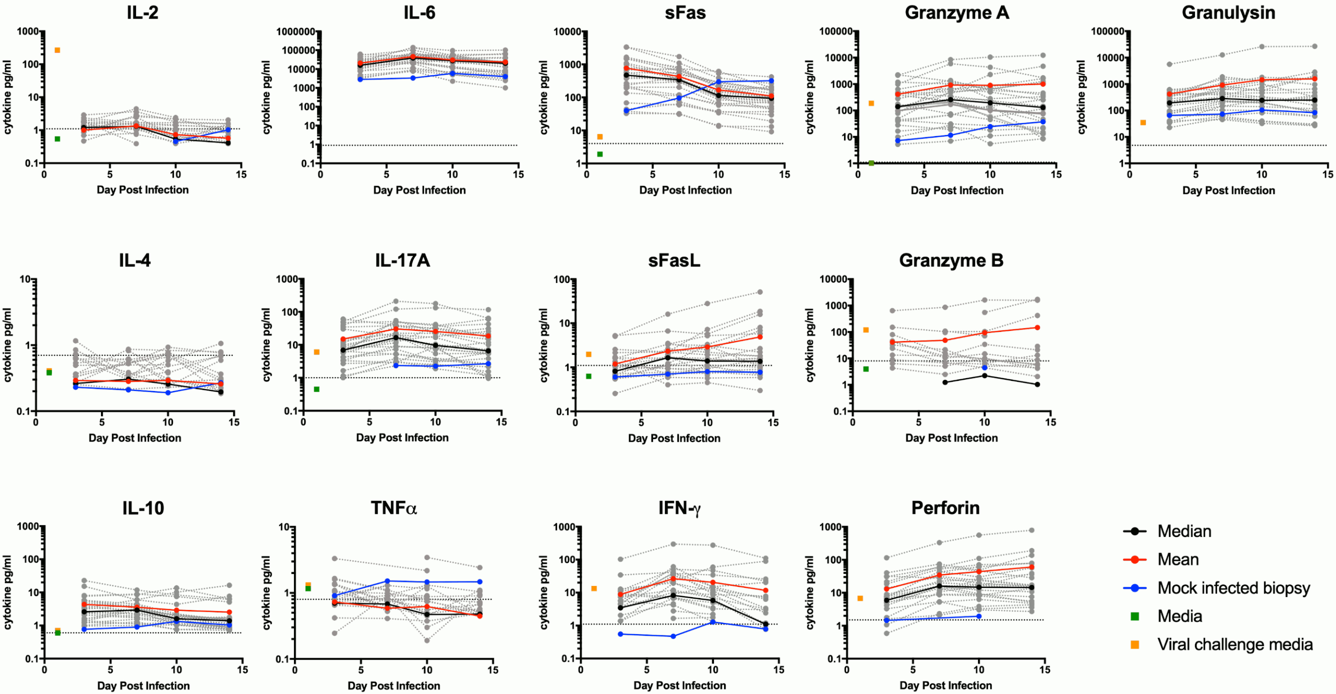

Supplement: Supplementary file 2 — Supplementary Figure S1. [file 41598_2020_76976_MOESM2_ESM.pdf]
